# Social Capital and Subjective Health Perception among Older Community Residents in Japan

**DOI:** 10.31662/jmaj.2025-0161

**Published:** 2025-09-05

**Authors:** Yasuko Sumitani, Takashi Tatsuse, Masaaki Yamada, Michikazu Sekine

**Affiliations:** 1 Department of Epidemiology and Health Policy, Faculty of Medicine, University of Toyama, Toyama, Japan

**Keywords:** social capital, social participation, subjective health perception, lack of confidence in health, neighborhood characteristics, Japan

## Abstract

**Introduction:**

Poor subjective health perception is a well-known risk factor for morbidity and mortality. This study aimed to investigate the relationship between social capital (SC) and health confidence among older individuals in a community setting.

**Methods:**

The study included 884 individuals aged 60-79 years residing in a city in the Hokuriku region (460 men and 424 women; mean age: 70.32 ± 4.03 years). The analysis considered the following variables: residential area, sex, age, presence or absence of cohabitants, SC (general trust in the community, reciprocity among community members, attachment to the region, and engagement in hobbies and other community activities), health counseling-related resources, and lack of health confidence. The multivariate logistic regression analysis used lack of health confidence as the dependent variable. Multiple models were constructed.

**Results:**

Of the 884 participants who provided data for the analyses, 176 (19.9%) reported a lack of confidence in their health. Women and individuals aged 70-79 years had significantly higher odds ratios (ORs) for reporting lack of health confidence (OR: 1.78 and 1.60, respectively). In men, the odds for reporting lack of health confidence were significantly higher among those who reported having no access to health or care counseling resources (OR 3.58, 95% confidence interval 1.47-8.72) and those who did not participate in sports-related group or club activities (OR 2.11, 95% confidence interval 1.15-3.86). In contrast, no significant associations were observed between SC factors and lack of health confidence among women.

**Conclusions:**

The findings suggest that, particularly among men, access to health counseling resources and participation in community activities contribute to greater confidence in own health.

## Introduction

In Japan, where the population is aging, extending healthy life expectancy has become an urgent issue, prompting the development of activities to promote health ^(1)^. The Japan Gerontological Evaluation Study (JAGES) and other reports have examined the relationship between healthy life extension, frailty prevention, and social participation ^(2), (3), (4), (5), (6)^. For example, Abe et al. ^(2)^ suggested that involvement in social activities, including participation in sports clubs/groups and neighborhood associations, is important in preventing physical frailty in older individuals. Sato et al. ^(3)^ also found that social activities decrease the likelihood of frailty, suggesting that community interventions are more beneficial in areas with fewer opportunities for civic participation and that insurer interventions compensate for a lack of social capital (SC).

Abe et al. ^(4)^ suggested that agricultural work, healthy daily activities, and social participation improve and prevent frailty and adverse events in rural areas in Japan. Noguchi et al. ^(5)^ found that the incidence of frailty diminishes with higher civic participation in a community, highlighting that promoting SC may help prevent frailty in older individuals in Japan. Xie and Ma ^(6)^ demonstrated that, in China, the association between social participation and physical frailty in older individuals depends on the type, frequency, and variety of social activities.

SC has been discussed across various contexts ^(7), (8), (9), (10), (11), (12)^. In Japan, SC is translated as social relationship capital and consists of three elements: trustworthiness, reciprocity, and network. Previous studies have suggested that SC influences health and health behaviors ^(13), (14), (15), (16)^. Furthermore, studies on the relationship between SC and health have addressed both group- and individual-level characteristics ^(17), (18)^. Even within the same municipality, SC status at the elementary school district level has been shown to play a significant role in health outcomes, suggesting that SC should be considered in district-level interventions.

There has been considerable debate regarding the mechanisms and pathways linking SC to health. In addition, various networks in each district contribute to SC and are intertwined with health and medical care. Hence, it is necessary to consider the complex and interrelated district-specific factors when designing interventions.

This study aimed to clarify the relationship between subjective health perception (i.e., confidence in one’s health) and SC, such as the presence of community activities and health counseling resources.

## Materials and Methods

### Participant selection

The study used data from the “Survey on Awareness of Connection and Watching Over Local People,” conducted in City A in the Hokuriku region using a self-administered questionnaire. This survey, funded by the Ministry of Internal Affairs and Communications under its Strategic Information and Communications Promotion Program, was conducted between November 2017 and November 2018. Approximately 10% of the older individuals aged 65-74 years in the five districts of City A participated, facilitated by regional development associations. The survey was self-administered, unsigned, and collected individually by mail to ensure anonymity and voluntary participation. A letter accompanying the survey explained that responding to the form implied consent to participate.

To ensure the representativeness of the sample, the survey forms were distributed across 145 administrative districts, which are subdivisions of the five living areas in City A. Distribution was coordinated through the representatives of each administrative district.

Regarding sample size, particular attention was paid to ensure that the incidence rate of the less frequent outcome was at least 10 times the number of independent variables used in the analysis. This was done to ensure that the sample size was sufficient for the multiple logistic regression analysis.

Of the 1,063 individuals surveyed, data from 884 (83.2%) aged 60-79 years who completed all required items were included in the analysis. City A has a population of approximately 90,000 and an aging rate of ~30%. The city was formed in 2005 by merging one city, three towns, and one village and features diverse areas, including old urban areas, residential developments, industrial zones, rural areas, and port regions.

This study was conducted as two separate surveys, the first covering two regions and the second covering three regions. Approval was obtained from the Institutional Ethics Review Committee for each survey (Toyama College of Social Welfare H29-012, 1 November 2017; Toyama College of Social Welfare H30-013, 6 September 2018).

### Analysis of items

Basic demographic variables included residential area (five districts), sex (male, female), age (60-69 years, 70-79 years), and number of family members (single, other).

The subjective health perception was measured via the reported confidence in one’s own health on a 5-point scale (from 1 = confident to 5 = not confident, with 1-3 points indicating a higher level of confidence and 4-5 indicating lack of confidence). Subjective health status is considered a predictor of life expectancy and activities of daily living ^(19)^. This study’s specific question (“Are you confident in your health?”) reflects not only the current self-assessment but also expectations for the future based on past experiences. Takai ^(20)^ described confidence as encompassing four aspects: self-affirmation, the ability to build relationships, competence, and resilience. Therefore, this question also implicitly evaluates the ability to maintain own health, making it a valuable indicator for designing interventions to foster health care.

Feelings toward the local community were evaluated based on three items: “generally trustworthy,” “willingness to help others,” and “attachment to the local area.” Trust in local people was measured on a 5-point scale, from 1 = very trustworthy to 5 = not at all trustworthy, with a score of 1-2 indicating high trustworthiness and 3-5 indicating low trustworthiness. Willingness to help others was evaluated on a 5-point scale, from 1 = very much to 5 = not at all, with a score of 1-2 indicating high reciprocity and 3-5 indicating low reciprocity. Attachment to the region was assessed on a 5-point scale, from 1 = very attached to 5 = not at all attached, with scores 5 indicating strong and weak attachment, respectively.

Health counseling resources were categorized as follows: “1: places where advice can be sought casually,” “2: places where advice can be sought but not casually,” and “3: no places available.”

The frequency of participation in community activities, such as membership in clubs for older adults, volunteer groups, sports groups and clubs, hobby groups, study and cultural circles, and skill-sharing groups, was assessed on a 6-point scale: 1 = 4 or more times a week, 2 = 2-3 times a week, 3 = 1 time a week, 4 = 1-3 times a month, 5 = several times a year, and 6 = no participation. The action of “talk to individuals living alone” was evaluated on a 5-point scale, from 1 = every day to 5 = do not care, with 1-3 points classified as positive and 4-5 classified as negative responses.

The items on community activity status and feelings toward the community were set with reference to the health-related SC indicators proposed by the JAGES project ^(13)^.

### Statistical analysis

To examine the relationship between lack of confidence in one’s health and community activities and other parameters, we used chi-square tests for categorical variables based on attribute factors (χ^2^ tests).

Multivariate logistic regression analyses were conducted to investigate the association between SC and lack of confidence in health. Odds ratios (ORs) and 95% confidence intervals were calculated. Multiple models were constructed: Model 1 adjusted for residential area; Model 2 adjusted for residential area and demographic factors (sex, age, number of family members); Model 3 adjusted for Model 2 variables and social resources (availability of health counseling services); Model 4 adjusted for Model 3 variables and SC (trust in local people, willingness to help others, attachment to the region); and Model 5 adjusted for Model 4 variables and participation in social activities (membership in senior clubs, volunteer groups, sports groups and clubs, hobby groups, study and cultural circles, participation in activities to share special skills and experiences with others, and interactions with neighbors.) In addition, a similar analysis was conducted by sex to examine sex-specific characteristics.

All analyses were performed using SPSS Statistics 20. Statistical significance was set at p < 0.05.

## Results

Table 1 presents the participant characteristics by sex. Among the 884 individuals who provided data for the analyses, 176 (19.9%) reported a lack of confidence in their health. Women were significantly more likely than men to live alone, have access to facilities for health and nursing care consultations, participate in sports-related groups or clubs, participate in hobby-related group activities, engage in study and cultural circle activities, share special skills and experiences with others, talk to individuals living alone, and lack confidence in their health.

**Table 1.** Characteristics of Subjects by Gender.

Item		Whole		Male		Female		p-value
		n=884		n=460		n=424		
		n	%	n	%	n	%	
mean age ± SD		70.32±4.03		70.34±4.08		70.30±3.99		0.877
age 2 classification	60-69years old	378	42.8	196	42.6	182	42.9	0.924
	70-79years old	506	57.2	264	57.4	242	57.1	
living alone and other	living alone	72	8.1	26	5.7	46	10.8	0.005^**^
	other	812	91.9	434	94.3	378	89.2	
generally trustworthy	high	734	83.0	387	84.1	347	81.8	0.365
	low	150	17.0	73	15.9	77	18.2	
willingness to help others	high	469	53.1	245	53.3	224	52.8	0.898
	low	415	46.9	215	46.7	200	47.2	
attachment to the region	strongly	765	86.5	400	87.0	365	86.1	0.704
	weakl	119	13.5	60	13.0	59	13.9	
having a place to go for health and nursing care	can casually ask for advice	246	27.8	96	20.9	150	35.4	< 0.001^***^
	can ask for advice but not casually	509	57.6	279	60.7	230	54.2	
	no places	129	14.6	85	18.5	44	10.4	
belonging to a senior club	yes	598	67.6	320	69.6	278	65.6	0.204
	no	286	32.4	140	30.4	146	34.4	
volunteer groups	yes	447	50.6	232	50.4	215	50.7	0.935
	no	437	49.4	228	49.6	209	49.3	
sports groups and clubs	yes	478	54.1	225	48.9	253	59.7	0.001^**^
	no	406	45.9	235	51.1	171	40.3	
hobby groups	yes	442	50.0	188	40.9	254	59.9	< 0.001^***^
	no	442	50.0	272	59.1	170	40.1	
study and cultural circles	yes	315	35.6	132	28.7	183	43.2	< 0.001^***^
	no	569	64.4	328	71.3	241	56.8	
activities to share skills and experiences with others	yes	196	22.2	89	19.3	107	25.2	0.035^*^
	no	688	77.8	371	80.7	317	74.8	
talking to people who live alone	yes	642	72.6	319	69.3	323	76.2	0.023^*^
	no	242	27.4	141	30.7	101	23.8	
confidence in one’s own health	confident	708	80.1	381	82.8	327	77.1	0.034^*^
	lack of confidence in health	176	19.9	79	17.2	97	22.9	

*:p < 0.05　**: p < 0.01　***: p < 0.001 χ2 tests were performed for categorical data. t-test was performed for mean age.

Table 2 summarizes the factors related to lack of confidence in health across the entire population. Chi-squared tests revealed that sex (male < female), general trust in local people (high < low), attachment to the community (strong < weak), availability of health and care counseling (casual consulting < not casual consulting < no consulting), participation in sports-related groups or clubs (yes < no), and participation in hobby-related group activities (yes < no) were significantly associated with lack of confidence in health.

**Table 2.** Factors related to lack of confidence in health (Overall).

Item				not confident in one’s health																
		n	n	(％)	χ2 tests		model 1			model 2			model 3			model 4			model 5	
		884	176	(19.9)	*P* value	OR	(95%CI)	*P* value	OR	(95%CI)	*P* value	OR	(95%CI)	*P* value	OR	(95%CI)	*P* value	OR	(95%CI)	*P* value
residential area	A	159	27	(17.0)	0.315	1.00			1.00			1.00			1.00			1.00		
	B	172	44	(25.6)		1.68	(0.98-2.88)	0.058	1.68	(0.98-2.88)	0.059	1.72	(1.00-2.97)	0.051	1.65	(0.95-2.87)	0.074	1.57	(0.90-2.75)	0.114
	C	340	63	(18.5)		1.11	(0.68-1.83)	0.675	1.13	(0.68-1.85)	0.643	1.14	(0.69-1.89)	0.611	1.14	(0.68-1.89)	0.620	1.06	(0.63-1.79)	0.814
	D	112	22	(19.6)		1.20	(0.64-2.23)	0.575	1.24	(0.66-2.33)	0.502	1.24	(0.65-2.34)	0.517	1.21	(0.63-2.30)	0.567	1.21	(0.63-2.34)	0.571
	E	101	20	(19.8)		1.21	(0.64-2.29)	0.565	1.28	(0.67-2.45)	0.449	1.34	(0.70-2.58)	0.381	1.27	(0.65-2.45)	0.485	1.09	(0.55-2.14)	0.806
gender	male	460	79	(17.2)	0.034				1.00			1.00			1.00			1.00		
	female	424	97	(22.9)					1.42	(1.02-1.99)	0.041	1.41	(1.01-1.98)	0.044	1.58	(1.11-2.23)	0.011	1.78	(1.24-2.56)	0.002
age 2 classification	60-69years old	378	72	(19.0)	0.579				1.00			1.00			1.00			1.00		
	70-79years old	506	104	(20.6)					1.12	(0.80-1.57)	0.520	1.18	(0.84-1.67)	0.338	1.34	(0.94-1.91)	0.109	1.60	(1.09-2.33)	0.015
living alone and other	living alone	72	16	(22.2)	0.644				1.00			1.00			1.00			1.00		
	other	812	160	(19.7)					0.94	(0.52-1.70)	0.848	0.94	(0.52-1.70)	0.828	0.95	(0.52-1.74)	0.874	0.99	(0.54-1.82)	0.974
generally trustworthy	high	734	136	(18.5)	0.023							1.00			1.00			1.00		
	low	150	40	(26.7)								1.34	(0.83-2.14)	0.228	1.24	(0.77-1.99)	0.382	1.15	(0.71-1.87)	0.570
willingness to help others	high	469	85	(18.1)	0.157							1.00			1.00			1.00		
	low	415	91	(21.9)								1.05	(0.73-1.51)	0.795	1.00	(0.69-1.45)	0.988	1.00	(0.69-1.46)	0.997
attachment to the region	strongly	765	140	(18.3)	0.004							1.00			1.00			1.00	-	
	weakl	119	36	(30.3)								1.72	(1.06-2.80)	0.029	1.63	(1.00-2.67)	0.051	1.63	(0.99-2.68)	0.056
having a place to go for health and nursing care	can easily consult	246	40	(16.3)	0.000										1.00			1.00		
	can consult but not easily	509	95	(18.7)											1.22	(0.80-1.86)	0.357	1.25	(0.81-1.92)	0.320
	no places	129	41	(31.8)											2.59	(1.50-4.47)	0.001	2.35	(1.34-4.13)	0.003
belonging to a senior club	yes	598	109	(18.2)	0.070													1.00		
	no	286	67	(23.4)														1.18	(0.80-1.75)	0.401
volunteer groups	yes	447	80	(17.9)	0.152													1.00		
	no	437	96	(22.0)														0.90	(0.60-1.35)	0.600
sports groups and clubs	yes	478	77	(16.1)	0.002													1.00		
	no	406	99	(24.4)														1.56	(1.07-2.28)	0.020
hobby groups	yes	442	75	(17.0)	0.035													1.00		
	no	442	101	(22.9)														1.36	(0.92-2.02)	0.121
study and cultural circles	yes	315	54	(17.1)	0.135													1.00		
	no	569	122	(21.4)														1.15	(0.75-1.77)	0.524
activities to share skills and experiences with others	yes	196	35	(17.9)	0.478													1.00		
	no	688	141	(20.5)														0.85	(0.52-1.39)	0.525
talking to people who live alone	yes	642	121	(18.8)	0.219													1.00		
	no	242	55	(22.7)														1.11	(0.76-1.64)	0.585

model 1 to model5: Binomial logistic regression analysis (“I am not confident about my health” was used as the dependent variable, and the following variables were added sequentially as adjustment variables in models 1 to 5 and forced entry). model 1: residential area (5 areas from A to E). model 2: gender(male, female), age (60-69 years old, 70-79 years old), whether living alone or not (living alone, living with others). model 3:generally trustworthy (high, low), trying to be helpful (high, low), and attachment to the community (strong, weak). model 4: availability of a person or a place where they can easily consult about their health and care for themselves and their family members (some place they can easily consult, some place they can consult but not easily, and no place they can consult). model 5: Membership in a senior citizen club (yes/no), volunteer activities (yes/no), sports-related group or club activities (yes/no), hobby-related group activities (yes/no), study or culture circle activities (yes/no), activities to share their skills and experiences with others (yes, no), Talking to neighbors when they live alone (yes, no). OR: Odds ratio　95% CI: 95% confidence interval

The multivariate logistic regression analysis showed that women consistently had higher odds of lacking confidence in health across all models (OR: 1.42-1.78). Individuals in their 70s were more likely to lack health confidence than those in their 60s (Model 5, OR: 1.60). Lack of confidence in own health was reported at significantly higher rates among those with no attachment to the region (Model 3, OR: 1.72) or access to health care and counseling services (Models 4 and 5, OR: 2.35-2.59). Participation in sports-related groups and clubs was significantly associated with health confidence (Model 5, OR: 1.56).

Table 3 presents the factors associated with a lack of health confidence among men. The multivariate logistic regression analysis indicated that residing in District B was a significant predictor in all models, with ORs increasing as additional variables were included (Model 1 OR: 2.72; Model 2 OR: 2.74; Model 3 OR: 2.76; Model 4 OR: 2.77; Model 5 OR: 2.86). The absence of health and care consultation facilities was a significant factor in Models 4 and 5 (OR: 4.18 and 3.58, respectively). Non-participation in sports-related groups or clubs was significant in Model 5 (OR: 2.11).

**Table 3.** Factors related to lack of confidence in health (Males).

Item				not confident in one's health																
		n	n	(％)	χ2 tests		model 1			model 2			model 3			model 4			model 5	
		460	79	(17.2)	*P* value	OR	(95%CI)	*P* value	OR	(95%CI)	*P* value	OR	(95%CI)	*P* value	OR	(95%CI)	*P* value	OR	(95%CI)	*P* value
residential area	A	79	8	(10.1)	0.261	1.00			1.00			1.00			1.00			1.00		
	B	81	19	(23.5)		2.72	(1.11-6.65)	0.028	2.74	(1.12-6.72)	0.028	2.76	(1.12-6.82)	0.028	2.77	(1.11-6.95)	0.029	2.86	(1.11-7.40)	0.030
	C	175	29	(16.6)		1.76	(0.77-4.05)	0.182	1.78	(0.77-4.09)	0.177	1.79	(0.77-4.16)	0.174	1.85	(0.79-4.34)	0.155	1.90	(0.80-4.54)	0.149
	D	62	12	(19.4)		2.13	(0.81-5.59)	0.125	2.17	(0.82-5.76)	0.120	2.09	(0.78-5.61)	0.142	2.16	(0.80-5.86)	0.129	2.14	(0.76-5.98)	0.149
	E	63	11	(17.5)		1.88	(0.71-5.00)	0.207	1.92	(0.72-5.13)	0.195	2.07	(0.76-5.63)	0.152	2.01	(0.73-5.52)	0.178	1.67	(0.59-4.73)	0.338
age 2 classification	60-69years old	196	33	(16.8)	0.901				1.00			1.00		0.637	1.00			1.00		
	70-79years old	264	46	(17.4)					1.10	(0.67-1.81)	0.717	1.13	(0.68-1.87)	0.637	1.30	(0.77-2.20)	0.323	1.66	(0.94-2.91)	0.081
living alone and other	living alone	26	5	(19.2)	0.789				1.00			1.00			1.00			1.00		
	other	434	74	(17.1)					0.97	(0.35-2.69)	0.945	0.90	(0.32-2.52)	0.841	0.92	(0.32-2.64)	0.877	1.01	(0.34-2.97)	0.989
generally trustworthy	high	387	63	(16.3)	0.240							1.00			1.00			1.00		
	low	73	16	(21.9)								1.30	(0.61-2.78)	0.493	1.20	(0.56-2.59)	0.634	1.23	(0.56-2.68)	0.608
willingness to help others	high	245	42	(17.1)	1.000							1.00			1.00			1.00		
	low	215	37	(17.2)								0.81	(0.47-1.39)	0.438	0.79	(0.46-1.38)	0.415	0.72	(0.41-1.28)	0.268
attachment to the region	strongly	400	63	(15.8)	0.044							1.00			1.00			1.00		
	weakl	60	16	(26.7)								1.84	(0.88-3.84)	0.104	1.54	(0.73-3.25)	0.262	1.41	(0.66-3.03)	0.375
having a place to go for health and nursing care	can easily consult	96	9	(9.4)	0.000										1.00			1.00		
	can consult but not easily	279	44	(15.8)											1.84	(0.85-3.98)	0.121	1.88	(0.85-4.17)	0.121
	no places	85	26	(30.6)											4.18	(1.77-9.84)	0.001	3.58	(1.47-8.72)	0.005
belonging to a senior club	yes	320	49	(15.3)	0.139													1.00		
	no	140	30	(21.4)														1.08	(0.61-1.94)	0.786
volunteer groups	yes	232	31	(13.4)	0.035													1.00	
	no	228	48	(21.1)														1.05	(0.57-1.95)	0.876
sports groups and clubs	yes	225	25	(11.1)	0.001													1.00		
	no	235	54	(23.0)														2.11	(1.15-3.86)	0.015
hobby groups	yes	188	23	(12.2)	0.023													1.00		
	no	272	56	(20.6)														1.35	(0.72-2.53)	0.353
study and cultural circles	yes	132	16	(12.1)	0.076													1.00		
	no	328	63	(19.2)														1.04	(0.49-2.20)	0.914
activities to share skills and experiences with others	yes	89	11	(12.4)	0.212													1.00		
	no	371	68	(18.3)														1.01	(0.44-2.30)	0.982
talking to people who live alone	yes	319	47	(14.7)	0.044													1.00		
	no	141	32	(22.7)														1.36	(0.79-2.33)	0.269

model 1 to model5: Binomial logistic regression analysis (I am not confident about my health was used as the dependent variable, and the following variables were added sequentially as adjustment variables in models 1 to 5 and forced entry). model 1: residential area (5 areas from A to E). model 2: gender(male, female), age (60-69 years old, 70-79 years old), whether living alone or not (living alone, living with others). model 3: generally trustworthy (high, low), trying to be helpful (high, low), and attachment to the community (strong, weak). model 4: availability of a person or a place where they can easily consult about their health and care for themselves and their family members (some place they can easily consult, some place they can consult but not easily, and no place they can consult). model 5: Membership in a senior citizen club (yes/no), volunteer activities (yes/no), sports-related group or club activities (yes/no), hobby-related group activities (yes/no), study or culture circle activities (yes/no), activities to share their skills and experiences with others (yes, no), Talking to neighbors when they live alone (yes, no). OR: Odds ratio 95% CI: 95% confidence interval

Table 4 lists the factors associated with a lack of health confidence among women. The chi-squared tests revealed that regional attachment was the only significant factor, with weak attachment associated with a higher lack of confidence. However, the multivariate logistic regression analysis did not yield significant ORs for any variables across Models 1-5.

**Table 4.** Factors related to lack of confidence in health (Females)

Item				not confident in one's health																
		n	n	(％)	χ^2^ tests		model 1			model 2			model 3			model 4			model 5	
		424	97	(22.9)	*P* value	OR	(95%CI)	*P* value	OR	(95%CI)	*P* value	OR	(95%CI)	*P* value	OR	(95%CI)	*P* value	OR	(95%CI)	*P* value
residential area	A	80	19	(23.8)	0.762	1.00			1.00			1.00			1.00			1.00		
	B	91	25	(27.5)		1.22	(0.61-2.43)	0.579	1.22	(0.61-2.44)	0.568	1.29	(0.64-2.60)	0.479	1.24	(0.61-2.51)	0.553	1.21	(0.59-2.48)	0.611
	C	165	34	(20.6)		0.83	(0.44-1.58)	0.575	0.83	(0.44-1.58)	0.578	0.86	(0.45-1.66)	0.656	0.86	(0.45-1.67)	0.662	0.80	(0.41-1.57)	0.514
	D	50	10	(20.0)		0.80	(0.34-1.90)	0.618	0.82	(0.34-1.94)	0.647	0.83	(0.34-2.01)	0.680	0.80	(0.33-1.96)	0.631	0.85	(0.34-2.14)	0.735
	E	38	9	(23.7)		1.00	(0.40-2.47)	0.994	1.00	(0.40-2.49)	0.996	1.00	(0.40-2.54)	0.995	0.97	(0.38-2.47)	0.952	0.91	(0.35-2.36)	0.838
age 2 classification	60-69years old	182	39	(21.4)	0.561				1.00			1.00			1.00			1.00		
	70-79years old	242	58	(24.0)					1.16	(0.73-1.84)	0.544	1.28	(0.79-2.06)	0.313	1.38	(0.84-2.26)	0.202	1.60	(0.94-2.71)	0.081
living alone and other	living alone	46	11	(23.9)	0.853				1.00			1.00			1.00			1.00		
	other	378	86	(22.8)					0.95	(0.46-1.96)	0.892	0.97	(0.46-2.03)	0.938	0.98	(0.47-2.05)	0.950	0.97	(0.46-2.05)	0.937
generally trustworthy	high	347	73	(21.0)	0.071							1.00			1.00			1.00		
	low	77	24	(31.2)								1.38	(0.75-2.53)	0.304	1.32	(0.71-2.44)	0.380	1.21	(0.64-2.27)	0.561
willingness to help others	high	224	43	(19.2)	0.064							1.00			1.00			1.00		
	low	200	54	(27.0)								1.31	(0.80-2.16)	0.281	1.25	(0.75-2.08)	0.386	1.26	(0.75-2.12)	0.386
attachment to the region	strongly	365	77	(21.1)	0.044							1.00			1.00			1.00		
	weakl	59	20	(33.9)								1.68	(0.87-3.25)	0.124	1.71	(0.88-3.30)	0.114	1.82	(0.92-3.60)	0.084
having a place to go for health and nursing care	can easily consult	150	31	(20.7)	0.164										1.00			1.00		
	can consult but not easily	230	51	(22.2)											1.01	(0.59-1.71)	0.978	1.04	(0.61-1.79)	0.876
	no places	44	15	(34.1)											1.77	(0.79-3.96)	0.168	1.79	(0.77-4.13)	0.173
belonging to a senior club	yes	278	60	(21.6)	0.396													1.00		
	no	146	37	(25.3)														1.29	(0.75-2.22)	0.353
volunteer groups	yes	215	49	(22.8)	1.000													1.00		
	no	209	48	(23.0)														0.78	(0.45-1.37)	0.393
sports groups and clubs	yes	253	52	(20.6)	0.195													1.00		
	no	171	45	(26.3)														1.27	(0.76-2.13)	0.366
hobby groups	yes	254	52	(20.5)	0.158													1.00		
	no	170	45	(26.5)														1.35	(0.81-2.27)	0.250
study and cultural circles	yes	183	38	(20.8)	0.414													1.00		
	no	241	59	(24.5)														1.19	(0.69-2.07)	0.527
activities to share skills and experiences with others	yes	107	24	(22.4)	1.000													1.00		
	no	317	73	(23.0)														0.81	(0.43-1.51)	0.507
talking to people who live alone	yes	323	74	(22.9)	1.000													1.00		
	no	101	23	(22.8)														0.86	(0.49-1.51)	0.595

model 1 to model 5: Binomial logistic regression analysis (“I am not confident about my health” was used as the dependent variable, and the following variables were added sequentially as adjustment variables in models 1 to 5 and forced entry). model 1: residential area (5 areas from A to E). model 2: gender(male, female), age (60-69 years old, 70-79 years old), whether living alone or not (living alone, living with others). model 3: generally trustworthy (high, low), trying to be helpful (high, low), and attachment to the community (strong, weak). model 4: availability of a person or a place where they can easily consult about their health and care for themselves and their family members (some place they can easily consult, some place they can consult but not easily, and no place they can consult). model 5: Membership in a senior citizen club (yes/no), volunteer activities (yes/no), sports-related group or club activities (yes/no), hobby-related group activities (yes/no), study or culture circle activities (yes/no), activities to share their skills and experiences with others (yes, no), Talking to neighbors when they live alone (yes, no). OR: Odds ratio 95% CI: 95% confidence interval

## Discussion

Health confidence is crucial, as it reflects an individual’s outlook on maintaining health in the future. The results of the multivariate logistic analysis of all samples demonstrated significantly higher ORs for reporting lack of health confidence among women, those aged 70-79 years, those with weak regional attachment (Model 3), and those without access to health or care counseling resources (Models 4 and 5). When analyzed separately by sex, the ORs for reporting lack of confidence were significantly higher in men who had no access to health or care counseling resources or who did not participate in sports-related clubs or activities, whereas in women, no significantly increased ORs were observed for any of the examined factors.

### Sex differences

The multivariate logistic regression analysis across the entire sample indicated that women were significantly more likely to lack health confidence, with the strength of this association increasing as more variables were added. This highlights sex as a critical factor influencing subjective health perceptions. Previous studies have reported similar sex differences in the relationship between individual-level SC and subjective health perception and depression in older individuals ^(13)^, as well as in life expectancy from the perspective of subjective health perception ^(16)^. These findings suggest that sex differences play a key role in subjective health responsiveness.

The lack of significant ORs for women in the sex-specific analyses may reflect a tendency for women to remain socially active despite lacking confidence in their health. In contrast, men appear less likely to engage in social activities under similar circumstances. Ota et al. ^(14)^ reported that low SC is associated with poor subjective health perception and depressive tendencies and that there are sex differences in factors related to SC. They also reported that the association between subjective health perception and life expectancy is stronger in men ^(21)^.

Our findings suggest that men are more susceptible to the influence of regional conditions than women. In regional cities, men are more likely than women to continue living in the place they lived in as children, which may increase susceptibility to regional influences. Saito et al. ^(22)^ also highlighted the effects of sex and urbanity in a survey conducted as part of JAGES, emphasizing that these are important contributors to behavior.

### Differences in age

The effect of age increased as more variables were included, with significantly higher ORs observed for individuals aged 70-79 years than for those aged 60-69 years. This suggests that factors such as access to health and care counseling resources, community attachment, and social participation mitigate the impact of aging on subjective health perceptions. Mitoku et al. ^(16)^ found that social participation enhances activity (life ability) and physical and mental health, which correlate with subjective health perceptions. In the present study, social participation may have played a role in increasing activity and enhancing psychosomatic function, thereby mitigating the effects of aging.

### Differences in feelings toward the community

Based on the results from Model 3, individuals with weak attachment to the community were more likely to have poor subjective health perceptions. However, this effect diminished and became non-significant in Models 4 and 5. This suggests that a lack of access to health and care counseling resources and a lack of social participation strengthen the relationship between low attachment to the community and low subjective health perception. Previous studies have suggested that community attachment is more strongly influenced by the quality of one’s experience within the community than by the duration of residence ^(23)^. In the present study, lack of engagement within the community, such as lacking access to advice on health and care or social activities, was associated with low community attachment, leading to a diminished OR in Models 4 and 5.

### Differences in availability of health and nursing care consultation services

In Models 4 and 5, the absence of health and care counseling services was significantly associated with lower subjective health perceptions. The reduced OR in Model 5 suggests that lack of participation in social activities partially overlaps with this factor. A review by Kishi and Horikawa ^(24)^ highlighted that emotional support, having a larger network, and participation in social activities reduce the risk of early death and physical decline among older individuals, with stronger effects in men. The study also indicated that access to health and care advice is a key factor in the development of a health and care system. In the present study, access to health and care advice and participation in social activities were considered to play an important role in maintaining health.

### Differences in status of community activities

In Model 5, not participating in sports-related groups or clubs had a stronger negative impact on subjective health perceptions than participation. Previous studies, such as that by Shibata et al. ^(25)^, have indicated an association between participation in sports and improved subjective health. This study’s question regarding activities in sports-related groups or clubs likely captured various forms of sports engagement, including spectating, which has also been linked to enhanced quality of life in care facilities for older populations ^(25), (26), (27), (28)^. These findings suggest that people may not participate in sports-related groups or clubs not only because they lack confidence in their health but also because they lack access to sports-related resources. Establishing various types of sports-related resources and encouraging participation in and support for local sports teams may promote the health of local communities in the future.

### Significance, limitations, and future prospects

This study elucidated the relationship between subjective health perceptions and personal SC among residents in their 60s and 70s, with a focus on sex differences and the influence of specific regional contexts. However, due to the cross-sectional design, causal relationships could not be established. Furthermore, the survey population may be biased. Additionally, this analysis does not sufficiently control for key potential confounding factors that influence social participation and confidence in health, such as health status, socioeconomic factors, and educational background. Therefore, the results of this survey are limited in terms of generalizability.

Currently, the establishment of a community-based integrated care system is being promoted as a national policy in Japan ^(29)^. The system aims to maintain the dignity of older individuals and support their independent living by promoting the development of a comprehensive support and service provision system. This system enables older individuals to continue living in their familiar communities and lead meaningful lives until the end of life. Furthermore, the construction of this system is based on the autonomy and initiative of local communities, with the goal of tailoring care models to the specific characteristics of each region. The envisioned system is intended to evolve into a cyclical process, whereby individual residents’ activities foster social connections among residents, contribute to the development of personal SC, and ultimately strengthen and expand collective SC. This research aims to promote individual resident activities and contribute to healthier communities.

In the future, we would like to expand the scope of the study to other populations and conduct regional comparisons to enhance understanding of these relationships.

### Conclusion

This study explored the relationship between subjective health perception (i.e., confidence in one’s health) and personal SC, using the residential conditions of people in a specific region of Hokuriku. The results suggest that subjective health perceptions are influenced by the characteristics of the local area and that sex differences play a role in shaping various aspects of daily life. To reduce the number of individuals who lack confidence in their health, it is essential to focus on two main approaches: (1) foster a sense of attachment to the local area and (2) enhance confidence in local residents. Specifically, creating facilities where people can seek advice on health and care, promoting volunteer activities, encouraging participation in sports-related clubs and groups, and supporting hobby-related clubs and groups would be particularly important for men.

## Article Information

### Acknowledgement

This study used data from the “Regional Resident Awareness Survey,” conducted as part of the Strategic Information and Communications Research and Development Promotion Program titled “Research and Development of Community Development Using Advanced Sensing and Personal Information that Allows Elderly People Living Alone to Live Safely, Securely, and Comfortably” (FY 2017 and 2018). We would like to thank the residents who took part in the survey, the people who helped with the survey, those who offered valuable opinions, and Editage (www.editage.jp) for English language editing.

### Author Contributions

All authors contributed to the design of the study, critically reviewed the content, and approved the final version to be published.

### Conflicts of Interest

None

### IRB Approval Code and Name of the Institution

FukutanH29-012, 1 November 2017; FukutanH30-013, 6 September 2018 (Toyama College of Social Welfare). This study was conducted as two separate surveys, the first covering two regions and the second covering three regions. Approval was obtained from the institutional ethics review committee for each survey.
